# Targeting ROS/NF-κB signaling pathway by the seedless black *Vitis vinifera* polyphenols in CCl_4_-intoxicated kidney, lung, brain, and spleen in rats

**DOI:** 10.1038/s41598-021-96008-0

**Published:** 2021-08-16

**Authors:** Noha H. Habashy, Ahmad S. Kodous, Marwa M. Abu-Serie

**Affiliations:** 1 Biochemistry Department, Faculty of Science, Alexandria University, Alexandria, 21511 Egypt; 2 Radiation Biology Department, National Center for Radiation Research and Technology, Egyptian Atomic Energy Authority (EAEA), Cairo, Egypt; 3 Department of Medical Biotechnology, Genetic Engineering, and Biotechnology Research Institute, City of Scientific Research and Technological Applications (SRTA-City), New Borg EL-Arab, Alexandria, 21934 Egypt

**Keywords:** Cytokines, Diseases, Kidney, Brain injuries, Pharmaceutics, Toxicology

## Abstract

Carbon tetrachloride (CCl_4_) is an abundant environmental pollutant that can generate free radicals and induce oxidative stress in different human and animal organs like the kidney, lung, brain, and spleen, causing toxicity. The present study evaluated the alleviative mechanism of the isolated polyphenolic fraction from seedless (pulp and skin) black *Vitis vinifera* (VVPF) on systemic oxidative and necroinflammatory stress in CCl_4_-intoxicated rats. Here, we found that the administration of VVPF to CCl_4_-intoxicated rats for ten days was obviously ameliorated the CCl_4_-induced systemic elevation in ROS, NO and TBARS levels, as well as MPO activity. Also, it upregulated the cellular activities of the enzymatic (SOD, and GPx) and non-enzymatic (TAC and GSH) antioxidants. Furthermore, the gene expression of the ROS-related necroinflammatory mediators (NF-κB, iNOS, COX-2, and TNF-α) in the kidney, brain, and spleen, as well as IL-1β, and IL-8 in the lung were greatly restored. The histopathological studies confirmed these biochemical results and showed a noticeable enhancing effect in the architecture of the studied organs after VVPF intake. Thus, this study indicated that VVPF had an alleviative effect on CCl_4_-induced necroinflammation and oxidative stress in rat kidney, lung, brain, and spleen via controlling the ROS/NF-κB pathway.

## Introduction

Oxidative stress is defined as an imbalance between the reactive oxygen species (ROS) toxicity and the physiological antioxidant defense system that scavenges ROS or ameliorates the resulted damage^1^. The generated ROS that destroys all the cell aspects can yield lethal effects^1,2^ and plays a vital role in the pathogenesis of numerous human organs, including the brain, lung, liver, spleen, and kidney^3–5^. Consequently, decreasing the production of ROS is critical for preventing more oxidative damage and; for cell survival. Nevertheless, massive cellular damage causes removal of the damaged cell to maintain the surrounding healthy cells^6^. Various toxic chemicals such as methanol, carbon tetrachloride (CC1_4_), aromatic hydrocarbons, and bromobenzene can induce systemic organ injury by producing ROS^7,8^. The CCl_4_ was extensively used as a cleaner, solvent, and degreaser, both for home and industrial use. In addition, it is widely used as an experimental model to study the hepatotoxic effects of plant extracts and drugs, since the liver is the main organ exposed to CCl_4_ toxicity. Other organs can also be affected by its toxicity such as the kidney, lung, and spleen^9,10^.

Numerous traditional remedies have been studied in laboratory animal models for their protective capabilities against different chronic diseases, toxins, and toxic chemicals^11–14^. *Vitis vinifera* (VV) is one of the folk remedies' plants and functional foods, belongings to the Vitaceae family. It is a non-climacteric fruit and the most cultivated and consumed edible fruit in the world. VV has various health benefiting effects like its immune-enhancing, anti-inflammatory, neuroprotective, antioxidant, anti-apoptotic, and anti-carcinogenic properties. These promoting effects are related to its functional constituents, which include minerals, amino acids, proteins, sugars, organic acids, vitamins, and phytochemicals (flavonoids, carotenoids, tannins, anthocyanins, and proanthocyanins)^2,15,16^.

The protective power of different types and parts of VV such as stems, leaves, seeds, and juice against diverse hepatotoxic agents, including CCl_4_ has been examined^3–5,17^. In addition, our recently published work investigated, for the first time, the hepatotherapeutic potential and synergistic activity of the extracted seedless (both pulp and skin) black VV polyphenols (VVP) against CCl_4_-induced hepatotoxicity^2^. Fortunately, no previous study has attempted to investigate the therapeutic efficacy of VVP against CCl_4_-induced systemic toxicity (kidney, lung, brain, and spleen toxicity) in rats, to compare their therapeutic values in these target organs. Therefore, the current study was conducted to investigate this point in addition to comparing the therapeutic potential of these isolated polyphenols in the four studied organs using the heatmap clusters plots. The effects of the black VVP on the oxidative damage and necroinflammation by which CCl_4_ induced systemic destruction, were also evaluated here. For an explanation of the results, the phytochemical content and the in vitro antioxidant activities of VVP were examined.

## Results

### Phenolic composition and antioxidant potential of VVPF

The present study found that the total phenolic content of VVPF was 58.387 ± 0.755 mg gallic acid eq. g^−1^ VVPF. In addition, this study reported the presence of 16.718 ± 1.858 mg catechin eq of flavonoids, 1.098 ± 0.342 mg quercetin (QR) eq of flavonols, and 3.467 ± 0.466 mg cyanidin-3-glucoside (Cy-3-glc) eq of anthocyanins in each 1 g of VVPF.

**Figure 1:**
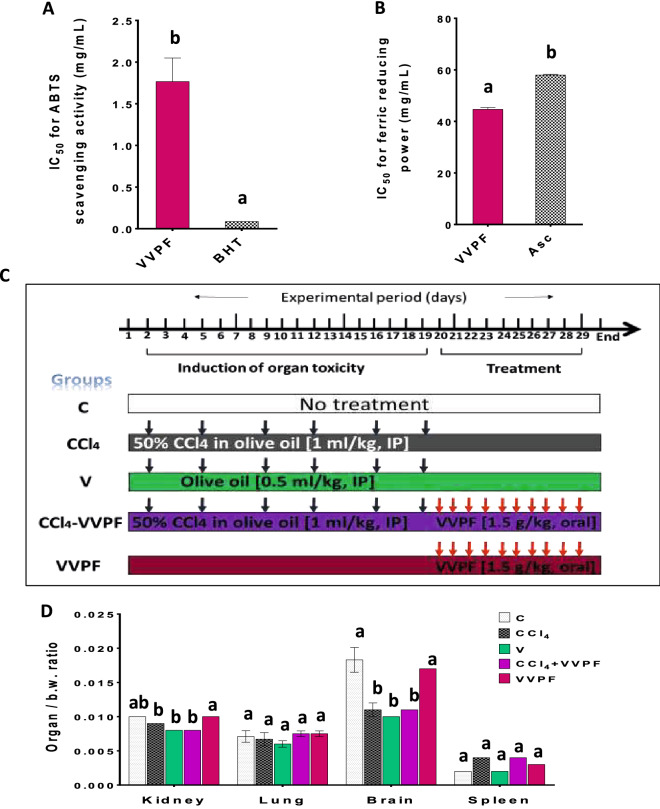
In vitro antioxidant activity of VVPF, experimental protocol with animal group treatments, and organ weights. (**A**) ABTS scavenging activity, (**B**) ferric reducing power (**C**) experimental protocol with animal group treatments. **(D)** organ weight/body weight (b.w.) ratio. Systemic toxicity was induced by CCl_4_ intraperitoneal (IP) injection for 3 weeks (twice/week). Animals in the CCl_4_ + VVPF group were gavage-fed VVPF for 10 days following systemic toxicity induction. The study comprises three control groups of animals, the untreated animals (C group), those gavage-fed VVPF for 10 days (VVPF group), as well as, animals that were injected with the olive oil (vehicle of CCl_4_, V group) for 3 weeks (twice/week). Different letters indicate the significance at *p* < 0.05. *Asc* ascorbic acid, *BHT* butylated hydroxytoluene, *IC50* 50% inhibitory concentration.

The VVPF showed a potent in vitro antioxidant activity with a total antioxidant capacity (TAC) value of 709.589 ± 25.939 mg Butylated hydroxytoluene (BHT) eq. g^−1^ VVPF. Moreover, it had potent ferric reducing power and was able to scavenge 2,2′-azino-bis (3-ethylbenzothiazoline-6-sulfonic acid (ABTS) radical (Fig. 1A, B). The results also showed that the ferric reducing power of VVPF was significantly (*p* < 0.05) higher (lower IC50 value "50% inhibitory concentration") than that of ascorbic acid (Asc), while its ABTS scavenging power was lower (higher IC50 value) than that of BHT.

### The influence of VVPF on CCl_4_-induced systemic toxicity

The VVPF exhibited a potent ameliorating impact on the CCl_4_-induced toxicity in kidney, lung, brain, and spleen tissues. This was demonstrated by a substantial improvement in systemic oxidative stress and necroinflammation.

The results revealed that the organ wt/b.w ratio for the kidney, lung, and spleen tissues had a non-significant change between all groups. However, a significant (*p* < 0.05) decrease was detected in the brain wt/b.w. ratios of rats in CCl_4_ (39.891%) and V (45.355%) groups as compared to the control "C" group. However, there was a non-significant change in these ratios in rats of the CCl_4_ + VVPF group relative to the CCl_4_ group (Fig. 1D).

### The ameliorating impact of the VVPF on the CCl_4_-induced renal oxidative stress

**Figure 2:**
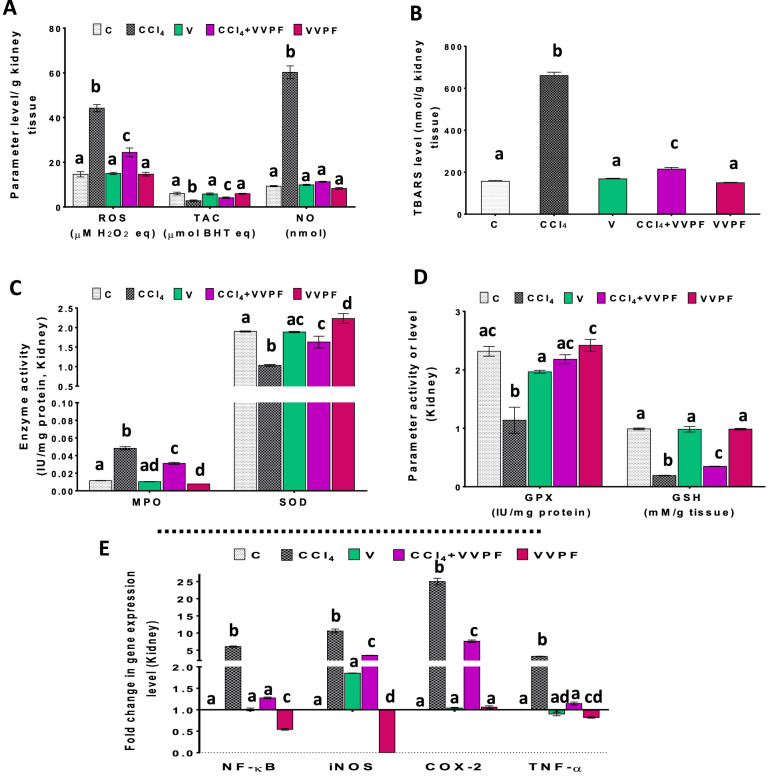
The improving activity of VVPF to CCl_4_-induced renal oxidative stress and necroinflammation.** (A)** reactive oxygen species (ROS), total antioxidant capacity (TAC), and nitric oxide (NO) levels **(B)** thiobarbituric acid reactive substances (TBARS) level **(C)** myeloperoxidase (MPO) and superoxide dismutase (SOD) activities **(D)** glutathione peroxidase (GPX) activity and reduced glutathione (GSH) level. **(E)** gene expression fold change of the pro-inflammatory mediators [nuclear factor-kappa (NF-κ)B, inducible nitric oxide synthase (iNOS), cyclooxygenase (COX)-2, and tumor necrosis factor (TNF)-α] in the kidney tissues. Results are expressed as mean ± S.E of 7 animals. *C* Control untreated rats; *CCl*_*4*_ rats with systemic toxicity induced by CCl_4_ injection (1 mL/kg b.w., IP, 6 times); *V* olive oil (vehicle of CCl_4_)-injected rats (0.5 ml/kg b.w., IP, 6 times); *CCl*_*4*_ + *VVPF* rats with systemic toxicity after their gavage-fed VVPF (1.5 g/kg b.w.) for 10 days. *VVPF* control rats gavage-fed only VVPF (1.5 g/kg b.w.) for 10 days. Different letters indicate the significance at *p* < 0.05; CCl_4_ + VVPF group was compared with the CCl_4_ group, while V and VVPF groups were independently compared with the C group. *BHT* butylated hydroxytoluene.

The data of this study revealed that the CCl_4_ induced oxidative stress in the kidney that was manifested by a significant (*p* < 0.05) elevation in the level of ROS (203.169%) and NO (545.833%). In addition, the thiobarbituric acid reactive substances (TBARS, 321.790%) level and the activity of myeloperoxidase (MPO, 321.579%) were increased in the CCl_4_ group, as compared to the C group (Fig. 2A–C). These results were accompanied by a massive decrease in the level of TAC (53.121%) and reduced glutathione (GSH, 80.647%) and the activity of the enzymatic antioxidants (superoxide dismutase "SOD, 45.727%" and glutathione peroxidase "GPX, 50.909%") as shown in Fig. 2A, C, D. However, there was a non-significant change in all of these oxidative stress markers in the kidney of rats in the V "vehicle" group (Fig. 2A–D).

The present study reported a good improvement in the oxidative stress parameters after administration of the VVPF for ten days (CCl_4_ + VVPF group). Hence the levels of ROS, NO, and TBARS and the activity of MPO were significantly (*p* < 0.05) reduced relative to that of the CCl_4_ group by 44.747%, 81.297%, 67.439%, and 35.362%, respectively (Fig. 2A–C). In contrast, the level of TAC (47.429%) and GSH (81.899%) and the activity of SOD (57.936%) and GPX (91.769%) were significantly (*p* < 0.05) upregulated in the kidney of the rats in this group (Fig. 2A, C, D). While the intake of VVPF alone for ten days (VVPF group) had a non-significant effect on all of the previous parameters, except for the MPO (decrease by 31.513%) and SOD (elevated by 17.413%) activities, which were significantly (*p* < 0.05) changed as related to the control group.

### The ameliorating impact of the VVPF on the CCl_4_-induced oxidative stress in the lung

**Figure 3:**
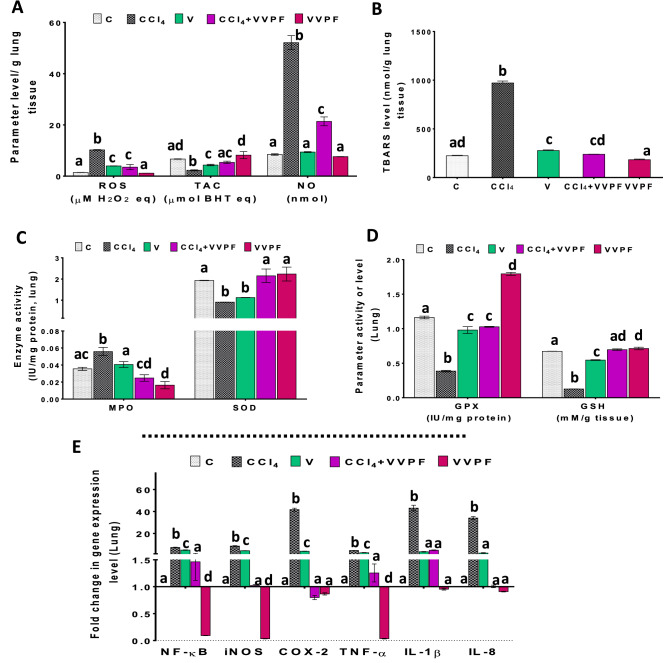
**The **improving activity of VVPF to CCl_4_-induced pulmonary oxidative stress and necroinflammation.** (A)** reactive oxygen species (ROS), total antioxidant capacity (TAC), and nitric oxide (NO) levels **(B)** thiobarbituric acid reactive substances (TBARS) level **(C)** myeloperoxidase (MPO) and superoxide dismutase (SOD) activities **(D)** glutathione peroxidase (GPX) activity and reduced glutathione (GSH) level. **(E)** gene expression fold change of the pro-inflammatory mediators [nuclear factor-kappa (NF-κ)B, inducible nitric oxide synthase (iNOS), cyclooxygenase (COX)-2, tumor necrosis factor (TNF)-α, interleukin (IL)-1β, and IL-8] in the lung tissues. Results are expressed as mean ± S.E of 7 animals. *C*, Control untreated rats; *CCl*_*4*_, rats with systemic toxicity induced by CCl_4_ injection (1 mL/kg b.w., IP, 6 times); *V*, olive oil (vehicle of CCl_4_)-injected rats (0.5 ml/kg b.w., IP, 6 times); *CCl*_*4*_ + *VVPF*, rats with systemic toxicity after their gavage-fed VVPF (1.5 g/kg b.w.) for 10 days. *VVPF*, control rats gavage-fed only VVPF (1.5 g/kg b.w.) for 10 days. Different letters indicate the significance at *p* < 0.05; CCl_4_ + VVPF group was compared with the CCl_4_ group, while V and VVPF groups were independently compared with the C group. *BHT* butylated hydroxytoluene.

In the lung, CCl_4_ also induced oxidative stress by elevating the levels of ROS, NO, TBARS, and the activity of MPO significantly (*p* < 0.05), by 131.883%, 516.092%, 332.458%, and 57.319%, respectively as compared to the control rats (Fig. 3A–C). Moreover, the level of TAC (65.779%), GSH (81.081%), as well as the activity of both SOD (53.195%) and GPX (66.797%) were greatly depleted in the lung after CCl_4_ injection (Fig. 3A, C, D). The results also found that the injection of rats with olive oil for 3 weeks (V group) led to a significant (*p* < 0.05) increase in the ROS and TBARS levels. Moreover, it substantially diminished the TAC and GSH contents in addition to the SOD and GPX activities, relative to the control (Fig. 3A–D). The percentages of these changes were 38.146%, 23.430%, 34.877%, 19.018%, 41.998%, and 15.715%, respectively.

Administration of VVPF for ten days (CCl_4_ + VVPF group) greatly ameliorated the disturbance in the pulmonary redox state that was induced by CCl_4_. This was achieved by a significant (*p* < 0.05) decrease in the ROS, NO, and TBARS levels, and MPO activity by 65.510%, 58.955%, 75.435%, and 55.496%, respectively, as related to the CCl_4_ group (Fig. 3A–C). However, the levels of TAC (136.084%) and GSH (445.627%), as well as the activity of SOD (137.817%) and GPX (166.154%) were significantly (*p* < 0.05) upregulated (Fig. 3A, C, D). While the administration of VVPF alone for ten days (VVPF group) not only had no damaging effect on the lung but also enhanced the pulmonary redox state. This has clearly appeared from its ability to enormously decrease the MPO activity (53.951%) and increase the GSH level (5.854%) and GPX activity (54.323%), as compared to the control untreated rats (Fig. 3C, D).

### The ameliorating impact of the VVPF on the CCl_4_-induced oxidative stress in the brain

**Figure 4:**
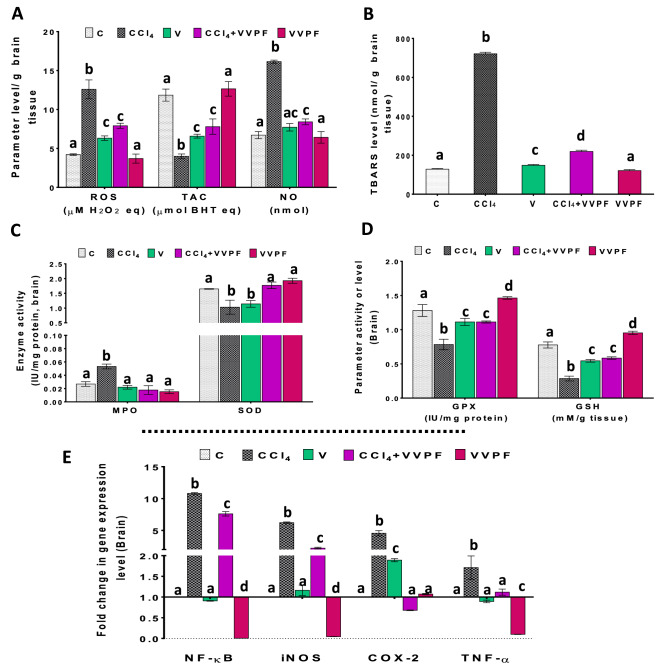
The improving activity of VVPF to CCl_4_-induced brain oxidative stress and necroinflammation.** (A)** reactive oxygen species (ROS), total antioxidant capacity (TAC), and nitric oxide (NO) levels **(B)** thiobarbituric acid reactive substances (TBARS) level **(C)** myeloperoxidase (MPO) and superoxide dismutase (SOD) activities **(D)** glutathione peroxidase (GPX) activity and reduced glutathione (GSH) level. **(E)** gene expression fold change of the pro-inflammatory mediators [nuclear factor-kappa (NF-κ)B, inducible nitric oxide synthase (iNOS), cyclooxygenase (COX)-2, and tumor necrosis factor (TNF)-α] in the brain tissues. Results are expressed as mean ± S.E of 7 animals. *C*, Control untreated rats; *CCl*_*4*_, rats with systemic toxicity induced by CCl_4_ injection (1 mL/kg b.w., IP, 6 times); *V*, olive oil (vehicle of CCl_4_)-injected rats (0.5 ml/kg b.w., IP, 6 times); *CCl*_*4*_ + *VVPF*, rats with systemic toxicity after their gavage-fed VVPF (1.5 g/kg b.w.) for 10 days. *VVPF*, control rats gavage-fed only VVPF (1.5 g/kg b.w.) for 10 days. Different letters indicate the significance at *p* < 0.05; CCl_4_ + VVPF group was compared with the CCl_4_ group, while V and VVPF groups were independently compared with the C group. *BHT* butylated hydroxytoluene.

Figure 4 demonstrates the ability of the CCl_4_ to induce imbalance in the cellular redox state of the rat brain. This was explained by the massive elevation in the cellular contents of ROS (198.719%), NO (140.580%), and TBARS (461.917%), and the activity of MPO (98.253%), as linked to the C group (Fig. 4A–C). Conversely, the TAC (66.333%) and the amounts of GSH (63.233%), along with the activity of SOD (37.463%) and GPX (38.788%), were drastically reduced when compared to the untreated control rats (Fig. 4A, C, D). The data also revealed the toxic effect of olive oil when it was administered for 3 weeks (V group). This toxicity was dictated by its influence on rising ROS (49.829%) and TBARS (15.612%) levels while declining TAC (44.444%) and GSH (29.822%) levels, as well as SOD (30.741%) and GPX (13.015%) activities when compared to the C group (Fig. 4A–D).

The administration of VVPF for ten days (CCl_4_ + VVPF group) was sufficient to improve the brain oxidative stress condition caused by CCl_4_ injection. After VVPF intake, the levels of ROS (37.175%), NO (47.879%), TBARS (69.551%), as well as the activity of MPO (66.516%) were dramatically diminished when related to the CCl_4_ group (Fig. 4A–C). In addition, the TAC and GSH levels, as well as the activities of SOD and GPX were significantly (*p* < 0.05) elevated when compared to the rats in the CCl_4_ group (Fig. 4A, C, D). The percentages of increase were 95.544%, 105.136%, 71.815%, and 41.951%, respectively. On the other hand, the intake of VVPF alone (VVPF group) had no toxic effects on the brain. In addition, VVPF was able to significantly (*p* < 0.05) increase the GSH level (22.809%) and the GPX activity (14.292%), relative to the control group as shown in Fig. 4D.

### The ameliorating impact of the VVPF on the CCl_4_-induced splenic oxidative stress

**Figure 5:**
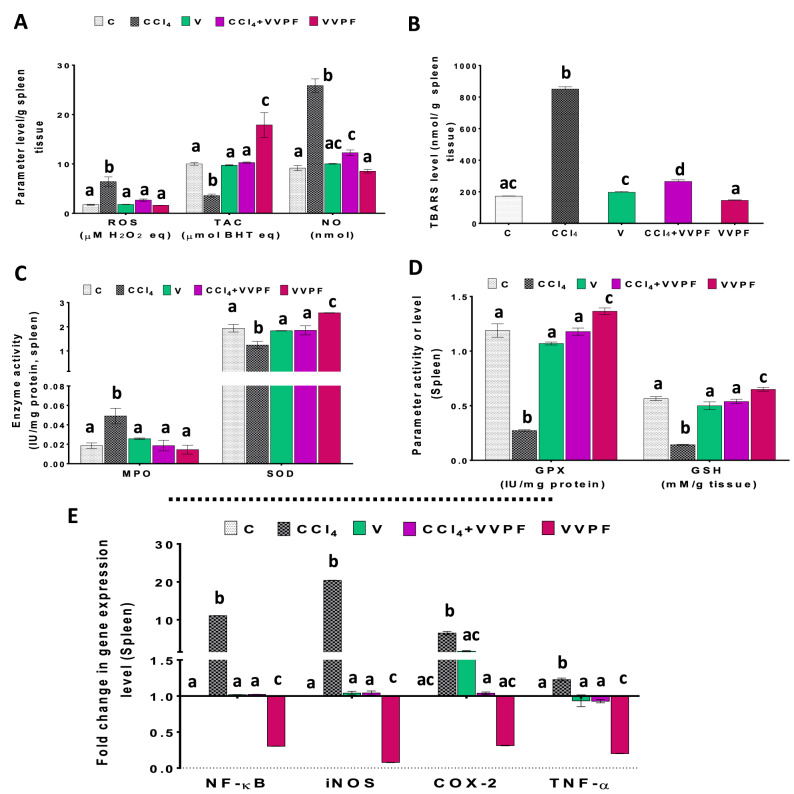
The improving activity of VVPF to CCl_4_-induced spleen oxidative stress and necroinflammation.** (A)** reactive oxygen species (ROS), total antioxidant capacity (TAC), and nitric oxide (NO) levels **(B)** thiobarbituric acid reactive substances (TBARS) level **(C)** myeloperoxidase (MPO) and superoxide dismutase (SOD) activities **(D)** glutathione peroxidase (GPX) activity and reduced glutathione (GSH) level. **(E)** gene expression fold change of the pro-inflammatory mediators [nuclear factor-kappa (NF-κ)B, inducible nitric oxide synthase (iNOS), cyclooxygenase (COX)-2, and tumor necrosis factor (TNF)-α] in the spleen tissues. Results are expressed as mean ± S.E of 7 animals. *C*, Control untreated rats; *CCl*_*4*_, rats with systemic toxicity induced by CCl_4_ injection (1 mL/kg b.w., IP, 6 times); *V*, olive oil (vehicle of CCl_4_)-injected rats (0.5 ml/kg b.w., IP, 6 times); *CCl*_*4*_ + *VVPF*, rats with systemic toxicity after their gavage-fed VVPF (1.5 g/kg b.w.) for 10 days. *VVPF*, control rats gavage-fed only VVPF (1.5 g/kg b.w.) for 10 days. Different letters indicate the significance at *p* < 0.05; CCl_4_ + VVPF group was compared with the CCl_4_ group, while V and VVPF groups were independently compared with the C group. *BHT* butylated hydroxytoluene.

Figure 5 elucidates the effect of the CCl4 injection on the cellular redox state of the spleen. In comparison to the control group, the levels of ROS (270.080%), NO (182.979%), and TBARS (395.516%) and the activity of MPO (165.852%) were markedly increased in the spleen of rats after 3 weeks of injection with CCl_4_ (Fig. 5A–C). Furthermore, the splenic TAC and the level of the non-enzymatic antioxidant (GSH), as well as the activities of the enzymatic antioxidant (SOD and GPX) were significantly (*p* < 0.05) decreased as compared to the C group (Fig. 5A, C, D). The percentages of this decrease were 64.035%, 74.867%, 35.894%, and 77.092%, respectively. However, no dramatic changes were observed in the spleens of rats after injection with olive oil (V group).

Our data found that the treatment with VVPF for ten days after CCl_4_ injection (CCl_4_ + VVPF group) showed great effects on the splenic oxidative stress. This was clearly inferred from the ability of VVPF to normalize most of the cellular redox state parameters. In this group, the levels of ROS, NO, TBARS, and the activity of MPO were significantly (*p* < 0.05) reduced relative to the CCl_4_ (Fig. 5A–C) by 58.276%, 52.519%, 68.816%, and 62.098%, respectively. In contrast, the levels of TAC and GSH and the activities of SOD and GPX were significantly (*p* < 0.05) increased (Fig. 5A, C, D) by 185.366%, 278.584%, 48.936%, and 332.962%, respectively. Concerning the VVPF group, the TAC and GSH levels, along with the SOD and GPX activities, were substantially enhanced in the spleens of rats that were gavage-fed VVPF for ten days (Fig. 5A, C, D) by 78.947%, 14.803%, 32.784%, and 14.872%, respectively. However, all other examined oxidative stress parameters (ROS, NO, TBARS, and MPO) were non-significantly (*p *˃ 0.05) affected in the spleen of these rats by 6.274%, 6.808%, 15.306%, and 21.574%, respectively.

### The anti-inflammatory influence of the VVPF in the kidney and lung tissues

The graphs in Figs. 2E, 3E elucidate the massive elevation of the pro-inflammatory mediators in kidney and lung tissues after CCl_4_ injection. Hence, the fold expression of nuclear factor-kappa (NF-κ)B, inducible nitric oxide synthase (iNOS), cyclooxygenase (COX)-2, and tumor necrosis factor (TNF)-α was upregulated in the kidney by 505.605%, 960.088%, 2399.110%, and 219.150%, respectively. Similarly, the fold expression of these mediators was dramatically increased in the lung tissue by 628.041%, 755.107%, 4066.674%, and 351.253%, respectively. Moreover, the fold expression of the interleukin (IL)-1β and IL-8 in lung tissue was substantially elevated (Fig. 3E) by 4207.250% and 3307.650%, respectively. Regarding, the rats which were gavage-fed olive oil for 3 weeks (V group), the fold expression of NF-κB, iNOS, COX-2, and TNF-α in the kidney was non-significantly changed when compared to the control group (Fig. 2E). Likewise, the fold expression of IL-1β and IL-8 in the lung tissue was non-significantly increased as related to the C group (Fig. 3E). In contrast, the fold expression of NF-κB (390.183%), iNOS (329.548%), COX-2 (286.581%), and TNF-α (148.410%) in the lung tissue was upregulated following the olive oil administration (Fig. 3E).

A great improving effect was observed for the VVPF (CCl_4_ + VVPF group) on the tested pro-inflammatory mediators induced in both kidney and lung following CCl_4_ injection. The fold expression of NF-κB and TNF-α in both tissues along with the iNOS, COX-2, IL-1β, and IL-8 were normalized (Figs. 2E, 3E). The % changes in the fold expression in the kidney relative to the CCl_4_ group were 78.928%, 64.111%, 66.921%, 69.514%, respectively. While these percentages in the lung tissue were 79.9158%, 72.199%, 88.049%, 98.082%, 88.860%, and 97.049%, respectively. Moreover, the fold expression of iNOS and COX-2 in the kidney was significantly (*p* < 0.05) reduced when compared to that of the CCl_4_ rats (Fig. 2E) by 66.921% and 69.514%, respectively. The rats in the VVPF group showed a significant (*p* < 0.05) ability to reduce the fold expression of the pro-inflammatory mediators in the kidney and lung tissues relative to the control group, especially for NF-κB, iNOS, and TNF-α. The percentages of reduction in the kidney were 45.702%, 99.993%, and 18.134%, respectively, while these values in the lung were 90.817%, 96.590%, and 96.438%, respectively. However, all other examined pro-inflammatory mediators either in the kidney (COX-2) or lung (COX-2, IL-1β, IL-8) were non-significantly (*p* > 0.05) changed (6.109%, 13.468%, 4.691%, and 9.194%, respectively).

### The anti-inflammatory influence of the VVPF in the brain and spleen tissues

Like in the kidney and lung tissues, the CCl_4_ injection caused upregulation in the pro-inflammatory mediators fold expression in the rat brain and spleen tissues (Figs. 4E, 5E). The fold expression of the NF-κB, iNOS, COX-2, and TNF-α was markedly upregulated in the brain by 979.924%, 522.286%, 355.513%, and 71.140%, respectively, and in the spleen by 1007.975%, 1937.934%, 549.203%, and 22.784%, respectively. However, the rats that were gavage-fed olive oil only for 3 weeks showed normal fold expression levels for all of the pro-inflammatory parameters in the spleen when compared to the control rats (Fig. 5E). The same results have been shown in the brain tissue, except for the fold expression of COX-2 that was upregulated significantly (*p* < 0.05), relative to the C group (Fig. 4E) by 89.064%.

Regarding rats that were administered VVPF for ten days after CCl_4_ injection (CCl_4_ + VVPF group), a dramatic decrease in the fold expression of NF-κB, iNOS, COX-2, and TNF-α in the brain and spleen, relative to the CCl_4_-intoxicated rats were reported. The percentages of this reduction in the brain tissue were 29.574%, 64.299%, 85.069%, and 34.672%, respectively. While these values in the spleen were 90.772%, 94.888%, 84.006%, and 24.426%, respectively. Similarly, the fold expression of NF-κB, iNOS, and TNF-α in the brain (99.376%, 95.678%, and 89.976%, respectively) and spleen (69.646%, 92.194%, and 79.936%, respectively) of rats in the VVPF group were markedly decreased in comparison to the control group. While the fold expression of COX-2 in both tissues did not change, relative to the control, after VVPF administration for ten days.

### Histopathological findings

**Figure 6:**
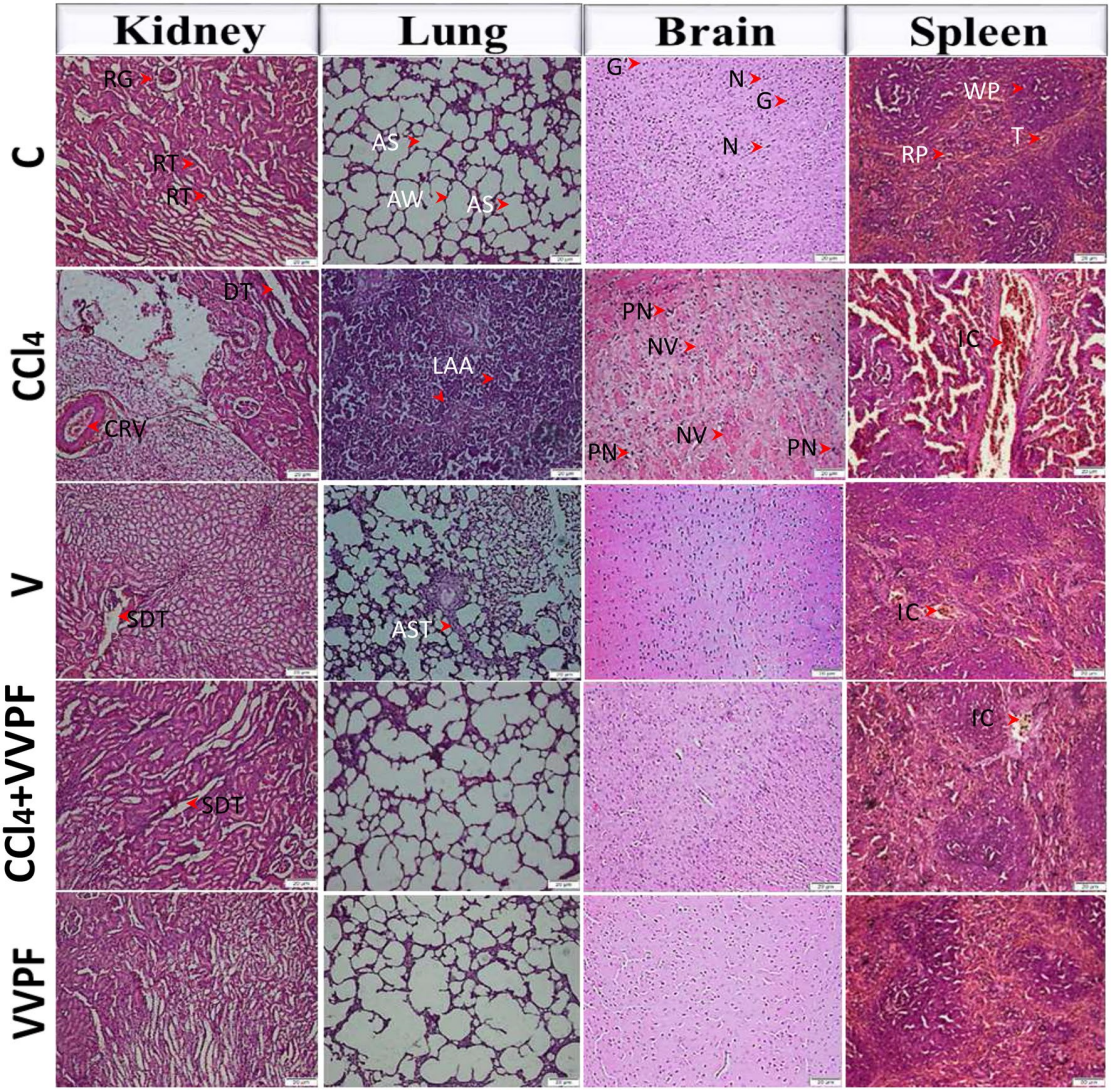
Photomicrographs (H&E stain, 20x) of kidney, lung, brain, and spleen specimens from the various study groups showed that VVPF alleviated the systemic toxicity caused by CCl4 injection. *AS:* alveolar septae, *AST*: alveolar septae thickness, *AW:* alveolar wall, *CRV*: congested renal vein, *DT*: degenerated tubules, *G*: glial cells, *IC*: inflammatory cells, *LAA*: loss of regular alveolar architecture, *N*: neurons, *NV*: neurons with the vacuole, *PN*: neuron with pyknotic nuclei, *RG*: *renal* *glomerulus,* *RP*: red pulp, *RT*: renal tubules, *SDT*: slight degeneration of tubules, *T*: trabecula, *WP*: white pulp.

The photomicrographs of the handled formalin-fixed pieces of the examined tissues showed the systemic toxicity of CCl_4_ and the improving influence of VVPF on the different studied organs (Fig. 6). The control sections of the kidney demonstrated the kidney cortex with normal renal tubules and glomeruli. However, the injection with CCl_4_ for 3 weeks showed severe toxicity that was manifested by congested renal vein (CRV (with infiltration of leucocytes and the renal tubules became degenerated and lost their lining epithelial cells and their typical appearance. These alterations revealed the necroinflammatory characteristics of this tissue. The administration of VVPF (CCl_4_ + VVPF) preserved the typical morphology of the kidney with only a slight degeneration of the tubules (SDT). Similarly, the olive oil injection (V group) demonstrated an SDT, however, no morphological changes were found in the kidney tissues of the VVPF group of rats.

Regarding the lung tissue, the control rats showed normal morphology of the alveolar sacs (AS) and alveolar walls. Severe damage was noticed in its architecture after CCl_4_ injection, which is characterized by loss of regular alveolar architecture, prevalent disorganised thickening of the alveolar septa, and alveolar space collapse. The administration of VVPF (CCl_4_ + VVPF) for ten days restored the lung morphology. Thickness in the AS with the alveolar air spaces narrowing was observed in the lung tissue pieces of the V group rats, while the morphology of lung tissue in the rats of the VVPF group showed no abnormal features.

The brain tissue in the control rats demonstrated normal structure with normal neurons and glial cells. After injection with CCl_4_, this typical architecture was altered to severe neurons degeneration with vacuoles and pyknotic nuclei in the neuronal cells. The gavage-fed VVPF for ten days relieved the CCl_4_ toxicity and preserved the brain morphology. Moreover, the rats of the V and VVPF groups showed normal morphology of the brain.

The spleen tissue of the control rats showed normal morphology with well-distinct red and white pulp sections. However, the spleen of the CCl_4_-intoxicated rats revealed massive damage in these regions with extreme inflammatory cells infiltration. This damage was mitigated after the administration of VVPF (CCl_4_ + VVPF), except for the minor influx of inflammatory cells. On the other hand, the rats injected with olive oil only (V group) had moderately unorganized white pulp regions with ambiguous regions and slight inflammatory cells recruitment. In contrast, administration of VVPF for ten days (VVPF group) had no adverse effect on animal spleen tissues, and the tissue showed well-defined white and red pulp compartments, as in the control group.

### The impact of CCl_4_ damage and VVPF therapeutic effects in the four examined organs

**Figure 7:**
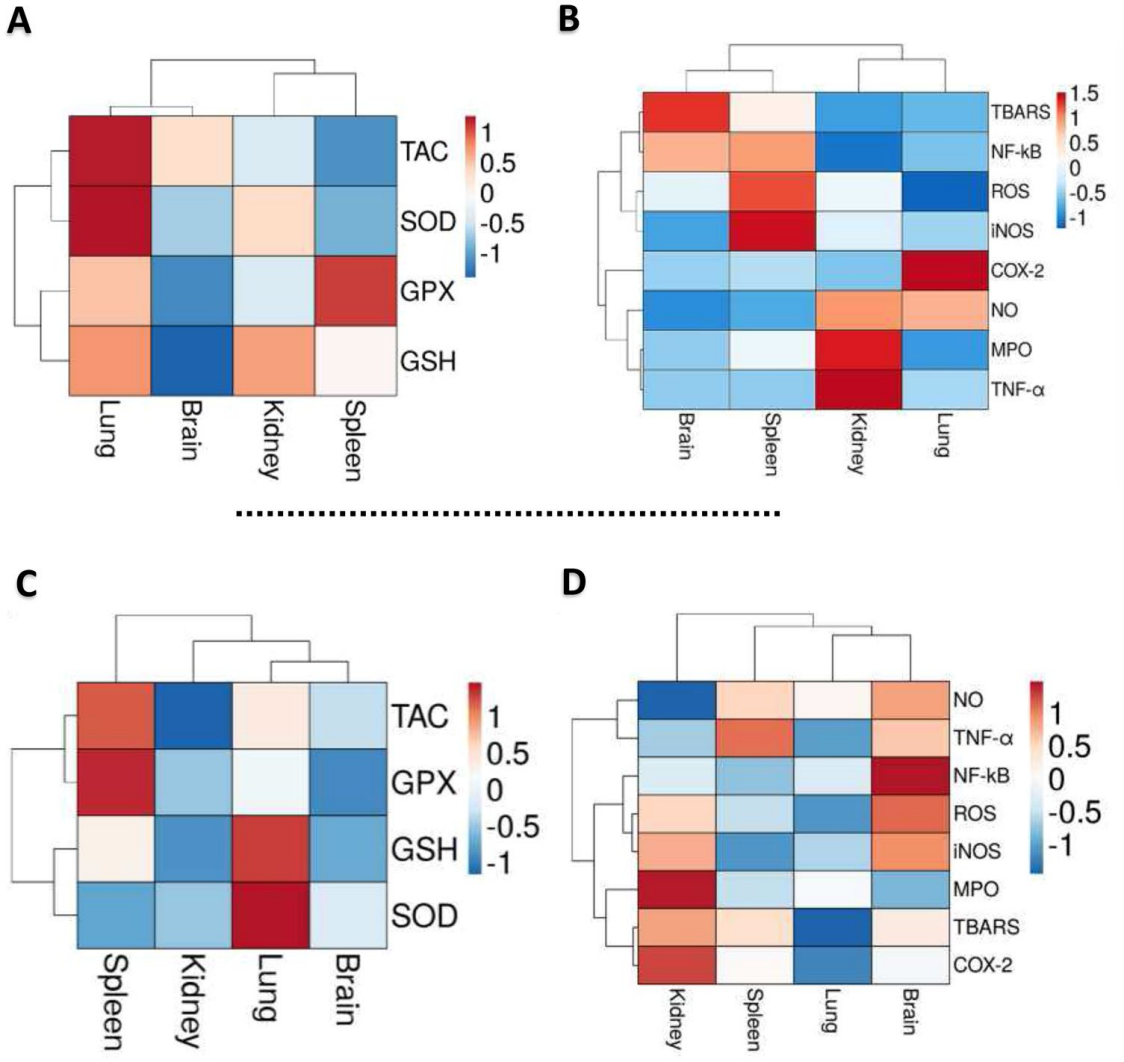
Heatmap diagrams for evaluation of the CCl_4_ damage effect and the therapeutic efficacy of VVPF in the target organs. The heatmaps clustered the considered oxidative stress and necroinflammation markers in the kidney, lung, brain, and spleen tissues of the rats in the CCl_4_ and CCl_4_ + VVPF groups. **(A)** Heatmap distribution of the CCl_4_-reduced markers in the CCl_4_ group compared to the control group. **(B)** Heatmap distribution of the CCl_4_-elevated markers in the CCl_4_ group compared to the control group.** (C)** Heatmap distribution of the up-regulated markers in the CCl_4_ + VVPF group compared to the CCl_4_ group. **(D)** Heatmap distribution of the down-regulated markers in the CCl_4_ + VVPF group compared to the CCl_4_ group. Results are shown as percentage change values relative to the control or the CCl_4_ group. The red color indicates the higher percentage increase values and the blue color refers to the lower ones. *COX-2*, cyclooxygenase-2; *GPX*, glutathione peroxidase; *GSH*, reduced glutathione; *iNOS*, inducible nitric oxide synthase; *MPO*, myeloperoxidase; *NF-κB*, nuclear factor-kappa B; *NO*, nitric oxide; *ROS*, reactive oxygen species; *SOD*, superoxide dismutase; *TAC*, total antioxidant capacity; *TBARS*, thiobarbituric acid reactive substances; *TNF-α*, tumor necrosis factor-α.

The influences of the CCl_4_ harmful effect and VVPF therapeutic effect in the four tested organs were evaluated by the heatmap plots (Fig. 7). The graphs in Fig. 7 cluster the studied oxidative stress and necroinflammation parameters in the rats treated with CCl_4_ (CCl_4_ group) and VVPF (CCl_4_ + VVPF group) in the different studied organs. The % increase of these parameters compared to the control (Fig. 7A, B) or CCl4 (Fig. 7C, D) group, respectively, was indicated by the color of the heatmap. The red color referred to a higher quantity of the parameter and the blue color referred to a lower one. Figure 7A, B demonstrates that the CCl_4_ can induce more oxidative stress and redox state disturbance in the spleen (higher ROS and lower TAC) followed by the kidney, brain, and finally lung tissue. Furthermore, as shown in Fig. 7B, the spleen and brain tissues had the highest fold expression of NF-κB, while the other mediators in the target organs had varying degrees of fold expressions.

On the other hand, Fig. 7C, D shows the highest ability of VVPF to improve the level of TAC and GSH in addition to the activity of SOD and GPX in the lung followed by spleen, kidney, and brain (Fig. 7C). In addition, this grape fraction was able to deplete the levels of the pro-inflammatory mediators, as well as ROS, NO, TBARS, and MPO in the lung followed by the spleen, kidney, and then the brain (Fig. 7D). Consequently, the heatmaps showed that the lung was the least affected tissue with the CCl_4_ damage and the most responsive tissue with the therapeutic values of VVPF.

## Discussion

Natural products derived from medicinal plants, herbal remedies, functional foods, and their ingredients have been used to treat a variety of diseases, including inflammation-mediated diseases^18^. VV is one of the medicinal remedies and functional foods that has various health benefits due to its rich functional constituents^2,15,16^. As an extension to our recently published research that reported the efficiency of the extracted VVPF on the CCl_4_-induced hepatotoxicity^2^, the current work evaluated its potency against CCl_4_-induced toxicity in kidney, lung, spleen, and brain (systemic toxicity). Thus we can compare its therapeutic influence on various organ toxicity to report the organ obtaining the most benefit from the therapeutic values of VVPF. The present study detected various types of phenolic compounds in VVPF, including flavonoids, flavonols, and anthocyanins. Therefore, this fraction exhibited a significant (*p* < 0.05) potent ferric reducing power more than that of Asc (Fig. 1B) besides its ability to scavenge the ABTS radical (Fig. 1A).

The CCl_4_ is extensively used in the development of experimental animal models of liver toxicity, as well as toxicity in other organs such as the kidneys, testis, brain, spleen, and lung^3^. This chemical toxin is metabolized in the liver by CYP2E1 and produces the reactive radical trichloromethyl (CCl_3_*), which combines with oxygen and is converted to another reactive radical, trichloromethyl peroxyl radical (CCl_3_OO*)^2,19^. The production of these radicals will increase the level of ROS in the liver leading to a disturbance of the organ redox state and induces oxidative stress, inflammation, fibrosis, and necroptosis and, in turn, hepatic damage^2,20,21^. This damaging effect in the liver will lead to changes and inflammatory response in the other organs (metabonomic alterations) such as the lung, kidney, and spleen^10,21,22^. In agreement with these previous studies, the current work reported the systemic toxicity of CCl_4_ by induction of massive redox state disturbance and gene expression of the pro-inflammatory mediators in the rat kidney, lung, brain, and spleen tissues. Hence the current study detected a substantial increase in the ROS level in these organs which was concomitant with a dramatic decrease in the redox state markers, TAC, GSH, GPX, and SOD, relative to those in the control group, after CCl_4_ injection. The high level of ROS, including the NO radicals in these tissues, will affect the cellular macromolecules, especially the membrane lipids, leading to their peroxidation (high TBARS level)^2,23^. This will lead to further ROS formation and accumulation in these examined rat organs, which resulted in the exhaustion of the GSH, GPX, and SOD^6,24^. Furthermore, the CCl_4_ injection caused a significant (*p* < 0.05) raising in the MPO activity that augmented the damage in the studied organs due to its essential role in the formation of hypochlorous acid (HOCl) in the neutrophils. Also, HOCl can interact with and consume the GSH in the presence of halide ions and H_2_O_2_, causing an elevation in lipid peroxidation and magnification of the oxidative stress condition^25–27^. These disorders led to a massive decrease in the cellular redox state (TAC) in all of the tested organs (Figs. 2, 3, 4, 5). Our results are in line with the previous works of Ali et al. and Shah et al.^28,29^ The heatmap plots (Fig. 7A, B) demonstrated that the lung tissue was the least affected tissue with the CCl_4_-induced redox state disturbance. Hence it had the least ROS level and the highest antioxidant indices, relative to those of the control rats, while the other examined organs revealed various responses to the CCl_4_ damage. This may be related to the route of administration, repeated doses are needed to generate more damage, or the portal circulation absorption^30^.

The present work interestingly disclosed mild toxicity on the lung and brain tissues after olive oil injection. These outcomes were in agreement with the previous study of Kouka et al., who found that the multiple intakes of this antioxidant-rich oil led to the consumption of the cellular antioxidant biomolecules, causing antioxidative stress in certain tissues^31^. The olive oil prooxidant activities in various organs can be attributed to the variations of the availability, concentration, persistence time, and distribution of its antioxidant compounds and metabolites in various rat organs^31,32^. This implies that these tissues did not exhibit valuable adaptations after ingestion of the antioxidants, and the reported adverse impact could be overturned if the olive oil was injected for a longer period^33^. Consequently, the CCl_4_, not the vehicle olive oil, was responsible for the systemic toxicity in the different organs of rats in the CCl_4_ group.

Our study reported that the gavage-fed VVPF1 for ten days to the CCl_4_-intoxicated rats restored the imbalance in the redox state by depleting the ROS level, suppressing lipid peroxidation, as well as normalizing most of the antioxidant indices, particularly in the lung and spleen (Figs. 2, 3, 4, 5). Our results are in agreement with the studies that reported the ameliorating role of food-rich in polyphenolic compounds to CCl_4_ toxicity, by reducing ROS, H_2_O_2_ and TBARS, in renal and pulmonary cells^34,35^. In addition, these outcomes were coherent with our in vitro results that confirmed the potent reducing power of VVPF and its ability in quenching ABTS radical. In addition, our recently published study reported the efficiency of this phenolic fraction in scavenging peroxide, superoxide, and hydroxide radicals^2^. This potency was related to the phenolic content of VVPF, including vanillic, gallic, caffeic, *p*-coumaric, syringic, ferulic, salicylic, and ellagic acids, along with the flavonoids and resveratrol^2^. These compounds have not only neutralized the ROS produced from CCl_4_ metabolism but also enhanced the antioxidant indices in all the studied organs^36–42^. The heatmap figures found that the improvement of the antioxidant defense system by VVPF in the lung tissue was higher than in other studied organs (Fig. 7C, D). This may be due to the ability of VVPF to improve the antioxidant parameters in the lung tissue more than other organs, as observed in the rats of the VVPF group. Hence, different antioxidant indices (TAC, SOD, GPX, GSH) in the target organs of rats in this group were substantially enhanced compared to the control, and the lung tissue showed a higher improving effect (Figs. 2, 3, 4, 5). In addition, the lung tissue was the least affected organ with the CCl_4_-induced oxidative stress (heatmap, Fig. 7A, B). Therefore, the lung tissue benefited the most from VVPF's antioxidant effects. The elevation of the GSH level by VVPF may be owed to the role of its constituents, particularly the flavonoids in boosting the gene expression of γ-glutamylcysteine synthetase, an essential enzyme for GSH synthesis^36^. In addition, previous studies have shown that grape peel polyphenols can recycle GSH from its oxidized form, GSSG, and keep GSH levels high^43^. The elevation of GSH level will result in a significant (*p* > 0.05) raising in the GPX activity due to its vital part as an enzyme co-substrate. Furthermore, resveratrol, one of the phenolic components of VVPF^2^, can induce GPX gene expression, as well as raise GSH levels^44^. All of these antioxidant influences will, in turn, lead to an elevation in the cellular TAC after VVPF administration^45^.

The present study also evaluated the effect of VVPF on the CCl_4_-induced necroinflammation in the kidney, lung, brain, and spleen tissues. The levels of pro-inflammatory mediators (Figs. 2, 3, 4, 5E), as well as the morphological changes (Fig. 6) in these target organs, were examined. The injection with CCl_4_ resulted in the elevation of the NF-κB, iNOS, COX-2, and TNF-α fold expression in all the tested organs. Furthermore, the fold expression of the pulmonary IL-1β and IL-8 was dramatically (*p* < 0.05) increased. The elevation in the ROS level in the different studied organs after CCl_4_ injection was the main cause for the induction of these inflammatory mediators. This can be accomplished by activating the NF-κB, which then upregulated the gene expression of the related pro-inflammatory mediators, including iNOS, COX-2, and TNF-α^46^. The morphological appearance of the kidney confirmed the occurrence of inflammation in the tissue, which was manifested by the CRV due to the infiltration of leucocytes. As a result, the renal tubules have degenerated and the renal epithelia were damaged (Fig. 6). Similarly, the lung tissue showed severe damage and thickening of the alveolar septa, which may be related to the elevation in the IL-8, IL-1β, and TNF-α. These cytokines are the key responsible mediators for the infiltration of the inflammatory cells in the lung tissue that, in turn, caused massive damage in the alveolar architecture^47^. In addition, these cytokines stimulated the production of a substantial level of NO that was reacted with superoxide radical to form the highly reactive peroxynitrite radical, causing nitrosative stress. As a consequence, the airway wall of the lung tissue thickened (Fig. 6), indicating mucosal inflammation and increased mucus glands, vessel area, and muscle mass, along with deposition of the connective tissue on the extracellular matrix^48^. Furthermore, IL-1β and TNF-α can activate the cellular necrosis and apoptosis pathways by altering the receptor and the ligand balance^9^. While the upregulation of the COX-twofold expression catalyzed the conversion of arachidonic acid to prostaglandin H2, along with the production of superoxide radicals that can augment the systemic damage induced by CCl_4_^6^. On the other hand, the elevated MPO activity boosted the CCl_4_-induced systemic inflammatory response by activating the neutrophils^26^. Therefore, the CCl_4_ injection to rats resulted in systemic necroinflammation via attraction of the inflammatory cells to the target organs (Fig. 6), as well as upregulating the fold expression of TNF-α to induce cell necrosis. These outcomes are in line with our previous study^2^.

On the other hand, the olive oil injection (V group) had no side effects on the kidney and spleen. However, it upregulated the gene expression of NF-κB, iNOS, COX-2, and TNF-α in the lung tissue and COX-2 in the brain tissue (Figs. 2, 3, 4, 5E). These negative effects may be due to these organs failed to adapt after consuming olive oil, as discussed above^33^. These results were in harmony with the histopathological findings that revealed inflammatory cells infiltration and moderate alveolar wall thickening in the lung tissue. Nevertheless, slight or no pathological changes were observed in the kidney, brain, and spleen of rats in this group (Fig. 6).

The treatment of CCl_4_-intoxicated animals with VVPF dramatically depleted and normalized the tested inflammatory mediators in the studied organs, especially in the lung tissue, compared to the CCl_4_ group. These results may be related to the antioxidant potential of VVPF and its vital role in reducing the ROS level, the main inflammatory inducer. Moreover, various anti-inflammatory active ingredients present in VVPF, including caffeic, ferulic, salicylic, p-coumaric, and ellagic acids^37^, as well as flavonoids^49^ and resveratrol^50^. Our previous study reported the synergistic anti-inflammatory effects between the VVPF polyphenols that may explain its great therapeutic role in the current study^2^. This data was consistent with the histopathological findings, which revealed an improvement in the morphology of the various examined intoxicated organs, particularly the lung tissue after VVPF administration (Fig. 6). Our results were in agreement with the previous studies that confirmed the anti-inflammatory effects of different grape extracts^19,51^.

The cluster heatmap plot (Fig. 7D) showed that the anti-inflammatory influence of VVPF appeared mostly in the lung. This may be due to the fact that this tissue was the least affected by CCl_4_ damage. Otherwise, the current study found that the VVPF showed considerable potency in reducing the pro-inflammatory mediators such as NF-κB, iNOS, and TNF-α in different organs when given alone to the non-intoxicated rats for ten days (Figs. 2, 3, 4, 5E). In addition, the architecture of the investigated organs has been well preserved as indicated by the histopathological study (Fig. 6). These results reflect the safety and potent anti-inflammatory activities of VVPF.

## Conclusions

The present work showed the systemic toxicity (kidney, lung, brain, and spleen) of CCl_4_ on rats and the great therapeutic influence of the seedless black VV fruit polyphenol-enriched fraction (VVPF). The biochemical and histopathological results clarified the role of VVPF in improving this toxicity by suppressing the ROS/NF-κB signaling pathway. Therefore, the systemic redox state disturbance and necroinflammation induced by CCl_4_ were improved. The potency of VVPF is owed to its phenolic compounds and their synergistic antioxidant and anti-inflammatory effects, as declared here and in our recently published work. Thus, VVPF is being proposed as a promising and effective natural anti-systemic toxicity agent for targeting ROS/NF-κB signaling pathways in different rat organs, particularly the lung tissue.

## Materials and methods

### Chemicals

BHT, Folin–Ciocalteau reagent, ABTS, CCl_4_, 2′,7′-dihydro-dichlorofluorescein diacetate (DCFH-DA) probe, TBA, tetra methoxy propane (TMP), GSH, O-dianisidine dihydrochloride (ODD), Asc and QR were provided from Sigma-Aldrich (St. Louis, MO, USA). Gene JET RNA purification kit, cDNA synthesis kit, and SYBR green master mix 2X kit were purchased from Thermo Fisher Scientific, USA. Forward and reverse primers were supplied from Bioneer, Korea. Total protein kit was bought from Biosystem, Spain. Other chemicals were obtained with a high grade.

### Preparation of VVPF

The Lebanese VV (NCBI:txid29760) was used for the enriched-phenolic fraction preparation as indicated in our previous study^2^. In brief, the seedless black fruit (pulp and skin) was ground with an electric grinder, and after lyophilization (Telstar, Terrassa, Spain), the powdered crude extract (yield, 14.020 ± 0.140 g/100 g grape) was obtained. Fifty grams of this powder were extracted twice for an hour using reflux with ethanol (70%, 500 mL for each, at 50 °C). Then the solution was filtered and freeze-dried to produce the final powdered fraction (VVPF), which was kept at − 20 °C until used.

### The polyphenolic contents of VVPF

The total levels of phenolics, flavonoids, flavonols, and anthocyanins in VVPF were quantified by spectrophotometric methods.

The levels of total phenolics in VVPF were determined by Folin–Ciocalteau reagent using the gallic acid calibration curve^52^. Total flavonoids were quantified using 10% aluminum chloride and 5% sodium nitrite. This method produced a yellow-colored complex with a maximum absorbance at 510 nm and the concentration of the total flavonoids was calculated using the catechin standard curve^53^. Total flavonol concentration was assessed at 440 nm using sodium acetate (50 g/L) and aluminum chloride (2%) solutions, as well as the QR calibration curve^54^.

The anthocyanins were determined by the pH-differential assay that depends on the reversible structural transformations of the pigments upon the pH change^55^. The VVPF was dissolved in two buffer solutions (pH 1.0 and pH 4.5), and the absorbance at 510 and 700 nm at each pH was measured. The VVPF's absorbance (A_VVPF_) was determined using the following equation: [A_VVPF_ = (A_510 _− A_700_)_pH 1.0 _− (A_510 _− A_700_)_pH 4.5_]. While the anthocyanins concentration was calculated as equivalent to Cy-3-glc from the next equation: [anthocyanins concentration = (A_VVPF_ × DF × MW × 1000)/(sample weight × ε)]. Where DF, MW, and ε is the sample dilution factor, the molecular weight, and molar absorptivity of Cy-3-glc, respectively.

### In vitro antioxidant activities of VVPF

The TAC, ferric reducing power, and anti-ABTS radical activity of VVPF were assessed in this study. The IC50 (50% inhibitory concentration) value for each assay was calculated by the GraphPad Instat program version 3.

The TAC of the VVPF was quantified using the reagent solution (28 mM sodium phosphate, 0.6 M H_2_SO_4_, and 4 mM ammonium molybdate). An aliquot of 900 µL of this reagent was mixed and incubated (at 95 °C for 90 min) with 100 µL of VVPF, ethanol (blank), or standard antioxidant (BHT); then the absorbance was recorded at 695 nm^56^.

Ferric reducing power was assessed by the Oyaizu method using potassium ferricyanide–ferric chloride reagent^57^. Different concentrations (0.06–1.00 mg/mL) of VVPF or Asc (standard antioxidant) were separately incubated (20 min, at 50 °C) with phosphate buffer (0.2 M, pH 6.6) and potassium ferricyanide (1%), followed by the addition of trichloroacetic acid (TCA, 10%). Afterwards, the solution was mixed with 1% ferric chloride and finally, the absorbance was recorded at 700 nm.

The ability of VVPF to scavenge and neutralize ABTS^+^ radical to ABTS was determined by the ABTS^+^ radical cation-decolorization method^58^. The ABTS^+^ radical (140 mM potassium persulphate was incubated with 7 mM ABTS at 25 °C, for 16 h) was incubated in dark for 5 min with serial concentrations (0.06–1.00 mg/mL) of VVPF or BHT (standard antioxidant). Then the absorbance of the remaining blue color was measured at 734 nm.

### Evaluation of the therapeutic effect of VVPF against CCl_4_-induced systemic toxicity

#### Animals and experimental protocol

In the current study, fifty male Albino rats (6 weeks, 140–200 g) were randomly divided into five groups (10 rats each) and they were purchased from MISR University for Science and Technology with pet welfare (assurance number: A5865-01). Animals had free access to tap water and regular animal feed and they were acclimatized for 2 weeks under ordinary conditions of temperature (about 30 °C) and a 12-h light–dark period. The experimental protocols and methodology of this work were approved by the Alexandria University Committee of Animal Care and Use and followed the Institutional Animal Care and Use Committee (IACUC) guidelines. The study was carried out in compliance with the ARRIVE guidelines.

Figure 1C describes the handling of animals in each laboratory group. Intraperitoneal (IP) injection of 50% CCl_4_ in olive oil (1 ml/kg b.w.) was given to rats every Sunday and Wednesday for 3 weeks to induce systemic toxicity^56,59^. The animals in the CCl_4_ + VVPF group were gavage-fed VVPF (1.5 g/kg b.w., dissolved in distilled water) every day for ten days. Rats were anaesthetized by inhalation of 2% isoflurane for 2 min, and immediately dissected at day thirty, then kidney, lung, brain, and spleen tissues were collected, washed (cold saline, 0.9% NaCl), and weighed. For the histopathological investigation, small pieces of each tissue were fixed in 10% formalin and the remaining tissues were kept at − 80 °C until they were used in the biochemical and molecular studies.

### Biochemical evaluation of the CCl_4_-induced systemic cellular redox state disturbance

The cellular redox state disturbance (oxidative stress) was evaluated in the studied tissues by determination of the intracellular ROS, TAC, NO, lipid peroxidation, and MPO. In addition, the enzymatic (SOD and GPX) and non-enzymatic (GSH) antioxidant parameters were quantified.

The tissue homogenates were prepared by separately homogenizing one gram of each studied tissue in cold phosphate buffer saline (PBS, 10 mL). Then the solution was centrifuged at 6000 rpm (4 °C) for 30 min to get the clear homogenates for the analyses.

#### Assessment of the intracellular ROS, TAC, lipid peroxidation, and NO levels

The ROS level was quantified using the highly sensitive DCFH-DA (5 μM) fluorescent probe and the H_2_O_2_ standard curve. In short, twofold diluted homogenate and the same amount of 1000-fold diluted DCFH-DA were mixed and incubated in dark at 37 °C for 5 min. Then the fluorescence intensity was recorded at 485 nm (excitation) and 520 nm (emission)^60^.

The ABTS radical cation method was used to determine the TAC levels in the tested tissues by mixing 20 µL of each tissue homogenate or PBS (control) or standard (BHT) with 2 mL of the radical solution^58^. The ABTS solution was prepared and the method was followed as described above in the in vitro assay. The percentage of inhibition was calculated, then the BHT standard curve was used to determine the TAC as BHT equivalent/g tissue of each studied organ.

The lipid peroxidation level was quantified spectophotometrically using TBARS method and the TMP standard curve^61^. While, Griess reaction was used to investigate the NO level at 490 nm. In this method, the tissue homogenate was reacted with Griess reagent (naphthylethylenediamine dihydrochloride "0.1%", phosphoric acid "2%", and sulfanilamide"1%") and sodium nitroprusside to produce bright-reddish-purple azo dye^62^.

#### Determination of MPO activity, enzymatic, and non-enzymatic antioxidant parameters

The MPO activity was determined colorimetrically using 1.2% H_2_O_2_ and 16.7 mg% ODD. The activity of MPO was expressed as μmoL of H_2_O_2_/min^63^. The activity of the enzymatic antioxidants (SOD and GPX) were determined followed the pyrogallol autooxidation^64^ and Rotruck^65^ methods, respectively. The enzyme specific activity (IU/mg protein) was calculated by dividing the value of enzyme activity by the protein content in each tissue homogenate, which was measured by the biuret method using the particular kit.

The non-enzymatic antioxidant (GSH) level was quantified using Ellman's reagent (5, 5′-dithio bis2-nitrobenzoic acid) and the GSH calibration curve. The produced yellow-colored complex was measured at 412 nm^66^.

### Molecular evaluation of the CCl_4_-induced systemic necroinflammation

The Quantitative real-time reverse transcription-polymerase chain reaction (qRT-PCR) technique was used to assess the necroinflammation in all the examined tissues. The current study investigated the NF-κB, iNOS, COX-2, and TNF-α gene expression to assess the necroinflammation in all the studied tissues. In addition, the critical pulmonary pro-inflammatory cytokines IL-1β and IL-8 were determined in lung tissue.

The total RNA was extracted using Gene JET RNA Purification Kit after homogenizing pieces of each studied tissue in lysis buffer containing β-mercaptoethanol. The lysate solution was centrifuged for 5 min at 14,000 rpm to obtain the supernatant for RNA extraction. Then the RNA was quantified and used for the cDNA Synthesis using the specific kit. The gene expression levels were examined by real-time PCR using the kit of SYBR green master mix and the primers of both the target and housekeeping (glyceraldehyde-3-phosphate dehydrogenase, GAPDH) genes (Supplementary Table 1). The fold expression of the tested genes was calculated by the comparative Ct method (number of threshold cycles at cross-point between amplification threshold and plot)^2^.

### Histopathological investigation

Following the standard histopathological protocol, the formalin-fixed tissue samples were embedded in paraffin wax then they were cut into 5 µm thickness slices and stained with hematoxylin and eosin. The phase-contrast microscope was then used to visualize the pathological aspects of the analysed tissues in all of the studied groups, and high-resolution pictures with 20× magnification were taken^2^.

### Statistical analysis

The data are expressed as mean ± SE and the significance was set at *p*-value < 0.05. A one-way ANOVA (one-way analysis of variance) using Duncan's analysis, measured the difference between the mean values of the analyzed groups. All data were tested for their regular distribution (skewness 0–0.953) prior to applying this parametric test. The study was conducted using SPSS program version 16 and the IC50 values for the in vitro antioxidant assessments were calculated using the GraphPad Instate software version 3.

## Supplementary Information

Supplementary Information.

## Abbreviations

ABTS: 2,2′-Azino-bis (3-ethylbenzothiazoline-6-sulfonic acid
Asc: Ascorbic acid
DCFH-DA: 2′,7′-Dihydro-dichlorofluorescein diacetate probe
IC50: 50% Inhibitory concentration
AS: Alveolar sac
BHT: Butylated hydroxytoluene
CCl_3_*: Trichloromethyl radicals
CC1_4_: Carbon tetrachloride
CCl_3_OO*: Reactive trichloromethyl peroxyl radical
COX-2: Cyclooxygenase-2
CYP2E1: Cytochrome P450
GSH: Reduced glutathione
GPX: Glutathione peroxidase
GAPDH: Glyceraldehyde-3-phosphate dehydrogenase
HOCl: Hypochlorous acid
iNOS: Inducible nitric oxide synthase
IACUC: Institutional Animal Care and Use Committee
IL: Interleukin
IP: Intraperitoneal
MPO: Myeloperoxidase
NF-κB: Nuclear factor-kappa B
ODD: O-dianisidine dihydrochloride
PBS: Phosphate buffer saline
QR: Quercetin
ROS: Reactive oxygen species
SDT: Slight degeneration of the tubules
SOD: Superoxide dismutase
TBARS: Thiobarbituric acid reactive substances
TMP: Tetra methoxy propane
TAC: Total antioxidant capacity
TNF-α: Tumor necrosis factor-α
VVPF: Vitis vinifera polyphenols fraction
