# Supplementary material for: Targeting ROS/NF-κB signaling pathway by the seedless black Vitis vinifera polyphenols in CCl4-intoxicated kidney, lung, brain, and spleen in rats
Source: Sci Rep. 2021 Aug 16;11:16575. doi: 10.1038/s41598-021-96008-0 (PMC8367948; doi:10.1038/s41598-021-96008-0)
Supplement: Supplementary file 1 — Supplementary Information. [file 41598_2021_96008_MOESM1_ESM.pdf]

Targeting ROS/NF-κB sigaling pathway by the seedless black *Vitis vinifera* polyphenols in CCl_4- intoxicated kidney, lung, brain, and spleen in Rats

Noha H. Habashy[1], Ahmad S. Kodous[2], and Marwa M.Abu-Serie[3]

[1]Biochemistry Department, Faculty of Science, Alexandria University, Alexandria, 21511, Egypt

[2]Radiation Biology Department, National Center for Radiation Research and Technology, Egyptian Atomic Energy Authority (EAEA).

. [3]Department of Medical Biotechnology, Genetic Engineering, and Biotechnology Research Institute, City of Scientific Research and Technological Applications (SRTA-City), New Borg EL-Arab, 21934, Alexandria, Egypt.

Supplementary Table 1: Forward and reverse primer sequences used in the qRT-PCR.

Primer name	Primer sequences(5′- 3′)
NF-κB	Forward 5′-TGCTAATGGTGGACCGCAA-3
	Reverse: 5′-CACTGCTTCCCGAATGTCTGA-3′
iNOS	Forward: 5′-ACCATGGAGCATCCCAAGTA-3′
	Reverse: 5′-CAGCGCATACCACTTCAGC-3′
COX-2	Forward: 5′-CCCATGTCAAAACCGTGGTG-3′,
	Reverse: 5′-CTTGTCAGGAATCTCGGCGT-3′
TNF-α	Forward:5′-GCCCAGACCCTCACACTC-3′
	Reverse: 5′-CCACTCCAGCTGCTCCTCT-3′
COL1A1	Forward: 5′- CATGTTCAGCTTTGTGGACCT-3′
	Reverse: 5′-GCAGCTGACTTCAGGGATGT-3′
TGF-β1	Forward: 5′- TGCTAATGGTGGACCGCAA-3′
	Reverse: 5′- CACTGCTTCCCGAATGTCTGA-3
IL-1β	Forward: 5′- GGGCCTCAAGGGGAAGAAGAATC-3′
	Reverse: 5′-ATGTCCCGACCATTGCTGTT-3′
IL-8	Forward: 5′- GAAGATAGATTGCACCGATG-3′
	Reverse: 5′- CATAGCCTCTCACACACATTTC-3′

NF-κB, nuclear factor-kappa B; ***iNOS***, inducible nitric oxide synthase; ***COX-2***, cyclooxygenase -2; ***TNF-α***, tumor necrosis factor-α; ***COL1A1***, collagen type I alpha one chain; ***TGF-β1***, transforming growth factor-β1; ***IL***, interleukin.
